# Neuroprotective Effects of Alpha-Lipoic Acid Against Behavioral Toxicity, Oxidative and Inflammatory Damage Caused by Titanium Dioxide Nanoparticles

**DOI:** 10.1007/s12011-025-04672-4

**Published:** 2025-06-02

**Authors:** Mahmoud A. Khedr, Sara E. El-Kazaz, Rashed R. Rashed, Hossam G. Tohamy, Mustafa Shukry, Amira A. Goma

**Affiliations:** 1 Department of Animal Husbandry and Animal Wealth Development, Faculty of Veterinary Medicine, Alexandria University, Alexandria, 21944 Egypt; 2 Department of Pathology, Faculty of Veterinary Medicine, Alexandria University, Alexandria, 21944 Egypt; 3 Department of Physiology, Faculty of Veterinary Medicine, Kafrelsheikh University, Kafrelsheikh, 33511 Egypt

**Keywords:** TiO₂-NPs, Alpha-lipoic acid, Neurotoxicity, Oxidative stress, Neuroinflammation, Neurobehavioral assessment

## Abstract

Titanium dioxide nanoparticles (TiO₂-NPs) are used widely in various industries, but emerging evidence suggests their ability to elicit neurotoxicity. The present study evaluated the alpha-lipoic acid (ALA) neuroprotective potential against TiO₂-NP-induced cognitive and molecular impairments in male Sprague–Dawley rats. Twenty-four rats were allocated into four groups: negative control, TiO₂-NPs (150 mg/kg, i.p.), ALA (50 mg/kg, orally), and TiO₂-NPs + ALA. Treatments were administered on alternate days for 28 days. Neurobehavioral tests, including the open field test (OFT), elevated plus maze (EPM), novel object recognition test (NORT), and Morris water maze (MWM), revealed that TiO₂-NPs impaired memory and increased anxiety-like behavior, while ALA co-treatment significantly restored behavioral performance. TiO₂-NPs exposure significantly decreased antioxidant enzymes (SOD, CAT, GSH), increased lipid peroxidation (MDA), elevated proinflammatory cytokines (TNF-α, IL-6), and apoptotic marker (caspase-3), and reduced neurotransmitter levels (GABA and dopamine). ALA administration reversed these alterations, indicating antioxidant, anti-inflammatory, and neuroprotective effects. Gene expression analysis showed TiO₂-NPs upregulated BAX, NF-κB, APP, and MAPT and downregulated BCL-2 and Nrf2, consistent with neurodegenerative and apoptotic signaling. ALA co-treatment normalized these gene expressions. Histopathological analysis confirmed structural damage in the cerebrum and cerebellum after TiO₂-NPs exposure, which ALA markedly improved. These findings suggest that ALA offers significant neuroprotection against TiO₂-NP-induced toxicity via its antioxidant, anti-inflammation, and anti-apoptosis properties, and supports being a potential protective agent against nanoparticle-induced neurodegeneration.

## Introduction

Nanoparticles are typically described as particles smaller than 100 nm. In comparison to larger particles, they exhibit greater permeability [1] and have distinct physical, mechanical, and chemical properties, which make them extensively used in biomedicine, pharmaceuticals, cosmetics, optics, and the food industry [2]. The extensive use of TiO_2_-NPs in various fields, such as dietary supplements, paints, toothpaste, sterilization processes, cosmetics, air purification, sunscreens, and treatment of wastewater [3], raised concerns about their potential cytotoxic effects [4]. Previous studies suggested that TiO_2_-NPs may cause neurobehavioral and neurotoxic impacts in living organisms [5]. This could be attributed to the fact that TiO_2_-NPs could induce oxidative damage, reduce the number of Nissl bodies, elevate the glial fibrillary acidic protein expression, influence glutamate and acetylcholine esterase activity, leading to hippocampal cell degeneration as reported by Song et al. [6] in mice. Further, it was reported that exposure to TiO_2_-NPs led to calcium deposition in nerve cells, promoted ependymal and glial cell proliferation, and disrupted the balance of trace elements, neurotransmitters, and enzymes in the brain of mice. These result in oxidative damage, apoptosis, impaired learning, and spatial recognition memory [7, 8]. The exposure of rats to TiO2-NPs triggers oxidative stress and inflammation, disrupts the blood–brain barrier, and reduces neuronal synaptophysin levels. Consequently, these raise the risk of neurological diseases with the increased usage of TiO2-NPs [9]. Titanium dioxide nanoparticles (TiO₂-NPs), widely used in food additives, cosmetics, and pharmaceuticals, have been shown to accumulate in tissues and induce toxicity. Experimental studies report that TiO₂-NPs impair mitochondrial function, generate reactive oxygen species (ROS), cause lipid peroxidation and DNA damage, and trigger apoptosis and inflammation [10]. In the kidneys, TiO₂-NPs upregulate injury and stress markers such as KIM-1, HSP-70, NF-κB, and TNF-α, while downregulating antioxidant defenses including Nrf2 and HO-1 [10].

ALA is an organosulfur compound produced from octanoic acid [11]. It is considered a natural antioxidant in mitochondria [12]. Alpha-lipoic acid (ALA) and its reduced forms, dihydrolipoic acid (DHLA), are thought to have antioxidant and neuroprotective effects due to their ability to cross the blood–brain barrier, scavenge free radicals, regulate the redox cycle, and reduce lipid peroxidation [13]. Furthermore, it acts as a cofactor in the mitochondria, preventing aging and dysfunction of the mitochondria [14]. It has been shown to help manage neurological disorders, including memory loss and cognitive dysfunction [15].

In addition, it protects against nerve degeneration [16] and reverses oxidative stress caused by cholinergic system dysfunction [17]. Therefore, the present study aimed to evaluate the neuroprotective effect of alpha-lipoic acid (ALA) against neurobehavioral toxicity induced by titanium dioxide nanoparticles (TiO₂-NPs) in male rats.

## Materials and Methods

### Chemicals

TiO_2_-NPs were purchased from Sigma-Aldrich Co., a company based in St. Louis, MO, USA, and ALA from Triveni Interchem Co. (India).

### Characterization of TiO_2_-NPs

TiO_2_-NPs were fabricated and characterized at the Nanotechnology Center, Chemistry Department, Faculty of Science, Kafrelsheikh University (Egypt), using scanning electron microscopy (SEM), X-ray diffractometer, and zeta potential/particle size analyzer. XRD measurements were performed with a Shimadzu 6000 X-ray diffractometer, using Cu Kα radiation (*λ* = 1.54056 Å). Scanning electron microscopy (SEM) with a JEOL (JSMIT100) operating at 30 kV assessed the surface morphology and particle size. Zeta potential data were gathered using a Brookhaven zeta potential and particle size analyzer [18].

### Preparation of TiO_2_-NPs and ALA

Distilled water was used to disperse TiO_2_-NPs, which were then sonicated for 5 min before use. ALA was dissolved in corn oil.

### Animals

Twenty-four Sprague–Dawley adult male rats, 3- to 4-month-old, with an average body weight of 160–180 g, were supplied by the Medical Research Institute, Alexandria University, Egypt. At the Medical Research Institute and Medical Technology Center for Research and Services, Alexandria University, Egypt, animal house rats were kept under the natural light/dark cycle. They were supplied with feed and water ad libitum. The commercial ration provided a broiler starter (Al-Eman Co., Egypt) containing 21% crude protein, 4.11% fat, and 2.44% crude fiber. The diet used met the NRC recommendations [19]. Rats were acclimatized for about two weeks before commencing the treatment.

### Experimental Design

Experimental procedures followed the Alexandria University Institutional Animal Care and Use Committee guidelines (ALEXU-IACUC, 120/2022). Twenty-four male rats were allocated randomly into four groups (six each): (1) negative control group obtained distilled water IP together with corn oil orally; (2) TiO_2_-NP-treated group administered TiO_2_-NPs (150 mg/kg bwt) IP; (3) ALA treated group given ALA (50 mg/kg bwt) orally; (4) TiO_2_-NP- and ALA-treated group was given both drugs. The treatment was administered for 28 days on alternate days [20, 21]. After the treatment, the rats'memory and learning ability were evaluated by neurobehavioral tests. Afterward, rats were humanely euthanized by decapitation, and brain samples were collected. The brain was split into two halves. One half was kept at − 80 °C for antioxidants, inflammatory biomarkers, and gene expression, while the other half was soaked in formalin solution for histopathological examination.

### Neurobehavioral Tests

#### Open Field Test (OFT)

This test assesses animals’ locomotor and exploratory behaviors. The open field arena comprises a square arena (40 L, 45 W, 45 H cm) divided into 16 equal squares by black grid lines. At the peripheral square, the rat was placed and permitted to explore for 5 min. Lines (N) were observed in both peripheral and central squares, rearing frequency, grooming (licking and scratching) frequency, excretion (defecation and urination) frequency, and time spent freezing. A thorough arena cleaning was performed at the end of the trial. The lower crossings and a higher rearing frequency are indicative of anxiety [22].

#### Elevated Plus Maze (EPM)

This test evaluates animals’ anxiety levels. The maze comprises two open arms (50 × 10 cm) and two closed arms of similar dimensions, with high side walls (40 cm). A central platform connects the arms, measuring 10 × 10 cm. A high edge about 0.5 cm surrounded the open arms, and the maze was 50 cm above the ground. Each rat was positioned in the central area, facing an open arm, and allowed 5 min to explore before being returned to its cage. An entry into the central region was considered when two paws were on it, while all four paws were considered for arm entry. Time elapsed to enter open or closed arms, time present in open and closed arms, open and closed arms entries (N), and time and entries in open and closed arms percent were recorded [23].

#### Novel Object Recognition Test (NORT)

Long-term memory recognition was assessed using the novel object recognition test in an open-field arena. Three objects, labeled A, B, and C, were used, where A and B are identical and C is different. The test was carried out over two (training and testing) days. During the training day, the rat was permitted to explore two similar objects (A and B) placed in the arena for 5 min. On the testing day, after 24 h, the novel object C replaced object B, alongside object A. The rat was again given 5 min to explore. A thorough arena cleaning was performed at the end of the trial. Approaches (*N*) to both objects and investigating time were recorded. Also, the discrimination ratio (*DR*) was estimated using the formula *DR* = (*N* − *F*)/(*N* + *F*), where *N* signifies the investigating time to the novel object and *F* symbolizes the exploring time of the familiar object, as described by [24].

#### Morris Water Maze (MWM)

The MWM analyzes spatial learning and memory. The apparatus consisted of a spherical tank (diameter of 120 cm and a height of 60 cm), with water filling to 45 cm depth maintained at 23 ± 1 °C. A non-toxic brown dye was applied to the water to obscure visibility. Four imaginary quadrants divided the tank (1, 2, 3, and 4), with the center of quadrant 3 (the target quadrant) having a hidden platform (5 cm in diameter) located 1–2 cm below the water ‘s surface. The test was conducted in two phases (place navigation and spatial probe), and it was performed on two consecutive days. All rats in all groups were tested on the same day for each phase. The first rats were taught to escape the hidden platform in the place navigation phase. Each rat was liberated into the water facing the tank wall and underwent four trials starting from a different quadrant in a clockwise sequence. The platform ‘s position remained fixed in the target quadrant for all trials. Each trial terminated when the rat reached the platform or after 60 s. If the rat did not locate the platform within 60 s, it was guided to it, allowed to remain on it for 5–10 s, and then returned to its home cage. The platform was removed in the spatial probe phase (second day). Each rat was liberated from the wall facing the target quadrant into the water and allowed to swim for 60 s before returning to its home cage. Time passed to locate the platform (escape), time needed to reach the target quadrant, expended time in the target quadrant, and the trials (*N*) to reach the target quadrant were recorded [22].

### Assessment of Oxidative and Antioxidative Markers

Catalase (CAT) activity was assessed using a Rat Catalase ELISA kit obtained from Biodiagnostic Co. (Dokki, Giza, Egypt; Catalog No. MBS2600683), which offers a detection range of 0.312–20 ng/mL and a sensitivity of 0.06 ng/mL. Superoxide dismutase (SOD) level in brain tissue homogenate was measured using a Rat SOD ELISA kit (Catalog No. CSB-E08555r) supplied by Cusabio Biotech Co., Ltd. (Houston, TX, USA), featuring a detection range of 7.8–500 U/mL and sensitivity below 1.95 U/mL To quantify reduced glutathione (GSH) in the brain, a Rat GSH ELISA kit (Catalog No. CSB-E12144r) from the same supplier was used, with a detection range of 7.8–500 ng/mL and sensitivity under 1.95 ng/mL Additionally, malondialdehyde (MDA) level was estimated using a Rat MDA ELISA kit (Catalog No. MBS268427) provided by MyBioSource, Inc. (San Diego, CA, USA), which has a detection range of 0.156–10 nmol/mL and a sensitivity of 0.05 nmol/mL.

### Evaluation of GABA and Dopamine in Brain Tissue

The gamma-aminobutyric acid (GABA) concentration in brain samples was quantified using a Rat GABA ELISA kit supplied by AssayGenie (Dublin, Ireland, Cat. No. RTFI01346), featuring a sensitivity of 18.75 pg/mL and a detection range of 31.25 to 2000 pg/mL Dopamine level in brain homogenate was evaluated using a rat-specific ELISA kit from CUSABIO (Houston, TX, USA, Cat. No. CSB-E08660r), with a sensitivity of less than 0.039 ng/mL and a detection range of 0.156–10 ng/mL.

### Evaluation of Apoptotic and Inflammatory Proteins in Brain Tissue

To analyze inflammatory and apoptotic proteins in brain tissue, tumor necrosis factor-alpha (TNF-α), interleukin-6 (IL-6), and caspase-3 were evaluated using ELISA kits. The corresponding kits were obtained from My BioSource (San Diego, CA, USA) under the catalog numbers MBS267737 (TNF-α), MBS764577 (IL-6), and MBS700575 (caspase-3). Each sample was analyzed in triplicate according to the procedures provided by the manufacturer.

### Real-Time PCR

According to the manufacturer’s guidelines, a Trizol reagent (iNtRON Biotechnology, Inc., Korea) was used to isolate total RNA from 100 mg of brain tissue. A NanoDrop spectrophotometer (UV–Vis, Q5000/Quawell, USA) was used to assess RNA concentration and purity. The extracted RNA was used to synthesize complementary DNA (cDNA) using the SensiFAST™ cDNA synthesis kit (Bioline, UK), following the manufacturer’s instructions. The synthesized cDNA samples were kept at − 20 °C until further investigation. Gene-specific primers and β-actin (used as an internal control) were designed based on accession numbers retrieved from GenBank (NCBI) (Table 1). Quantitative real-time PCR (RT-PCR) was performed to evaluate mRNA expression levels using the SYBR Green method (SensiFAST SYBR Lo-ROX kit, Bioline). The thermal cycling conditions included an initial denaturation at 95 °C for 10 min, followed by 40 amplification cycles of 95 °C for 15 s, 60 °C for 30 s, and a final extension step at 85 °C for 1 min. Relative gene expression levels were normalized to β-actin and calculated using the 2^ − ΔΔCT method [25].

**Table 1:** Primer sequences

Gene	Accession number	Forward primer (5′ → 3′)	Reverse primer (5′ → 3′)	bp
BAX	NM_017059.2	GGCGAATTGGCGATGAACTG	ATGGTTCTGATCAGCTCGGG	167
BCL2	NM_016993.1	GATTGTGGCCTTCTTTGAGT	ATAGTTCCACAAAGGCATCC	172
Nrf2	NM_031789	TGACCATGAGTCGCTTGCC	TCCTGCCAAACTTGCTCCAT	153
NF-κB	NM_001438066.1	GGCTCGGAAAGAAGTCGGAA	CCACAATGGAGGGGCTGAAT	185
APP	AY011335.1	TACATAGCCCCTTAGCCCGT	CACGTTCACACGAAGCATCC	110
MAPT	NM_017212.3	TCCTCGCCTCCTGTCGATTA	AGCTTGGTCCTCCATGTTCG	184
β-actin	NM_031144.3	CTGTGTGGATTGGTGGCTCT	AGCTCAGTAACAGTCCGCC	134

*BAX*, Bcl-2-associated X protein; *BCL2*, B-cell lymphoma 2; *Nrf2*, nuclear factor erythroid 2–2-related factor 2; *NF-κB*, nuclear factor kappa-light-chain-enhancer of activated B cells; *APP*, amyloid precursor protein; *MAPT*, microtubule-associated protein tau; *β-actin*, beta-actin

### Histopathological Examination

The second-gathered half of the brain tissue (cerebrum and cerebellum) was immediately fixed in 10% formalin for at least 24 h. Specimens were dried in ascending alcohol concentrations, cleared in xylene, and embedded in paraffin wax. Five-micron paraffin sections were stained with hematoxylin and eosin (HE) following Bancroft and Gamble [26]. The lesion’s severity was scored by estimating the affected area percentage in the entire section. Lesion score: (−) absence = 0%; (+) mild = 5–25%; (+ +) moderate = 26–50%; and (+ + +) severe = 50% of the examined tissue section.

### Statistical Analysis

The Statistical Package for Social Sciences software (SPSS, version 25) was used for statistical analysis. A principal component analysis determined the association between the measured parameters. A one-way general linear model (GLM) was applied to assess the effect of the treatment on the measured parameters. The findings are expressed as means ± standard error, with statistical significance determined at *P* < 0.05. A post hoc Duncan’s multiple range test was performed to discern treatment variations.

## Results

### Characterization of the TiO_2_-NPs

**Fig. 1:**
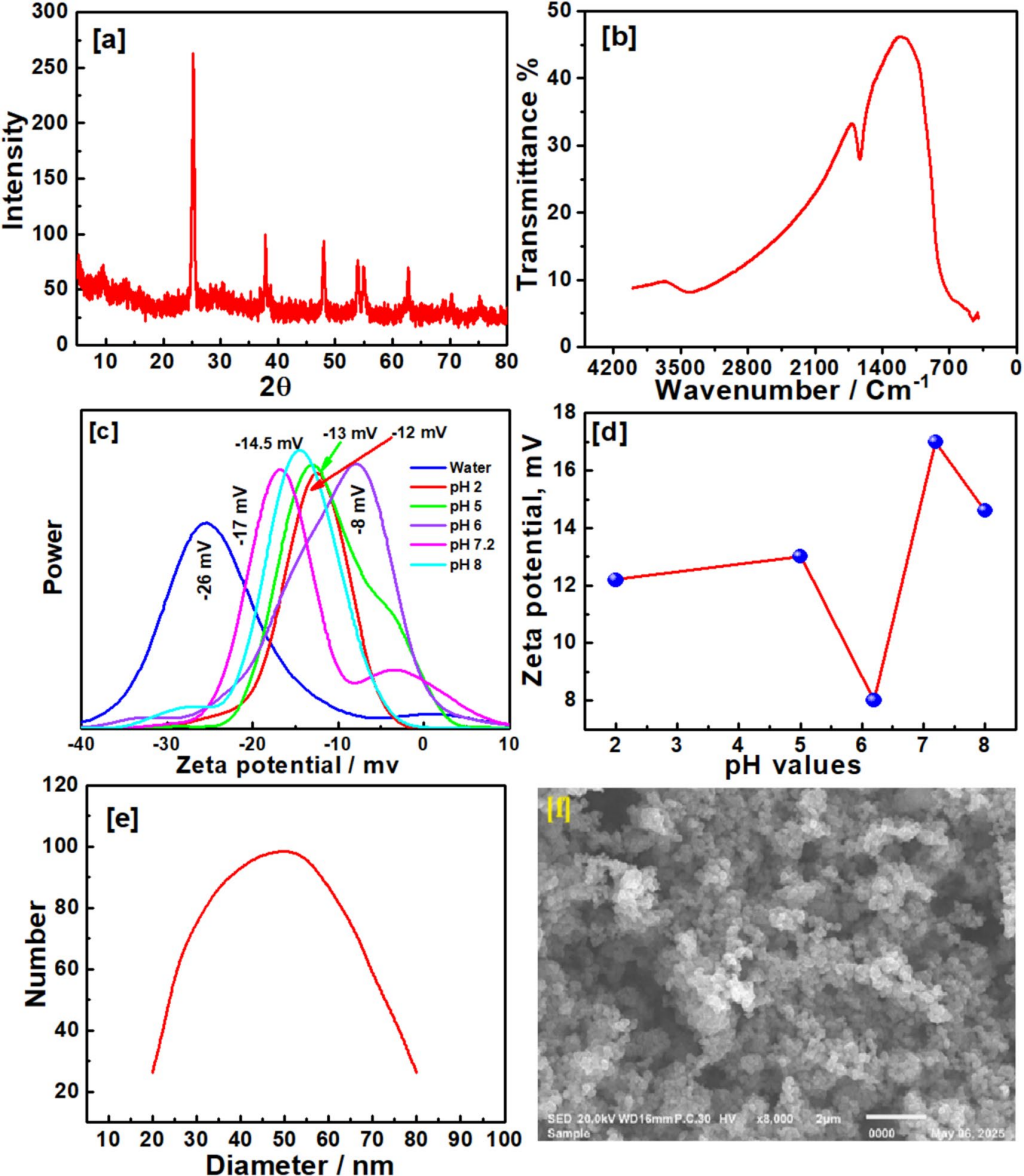
Characterization of the fabricated TiO2 NPS: **a** XRD patterns; **b** FT-IR spectra; **c**, **d** zeta potential at different pH; **e** dynamic light scattering (DLS); and **f** scanning electron microscopy

XRD analysis: No rutile or brookite phases were detected in the XRD pattern of the TiO₂ NPs (Fig. 1a), which corresponds to the usual diffraction peaks of the anatase phase (25.16°, 37.78°, 47.89°, 53.85°, 54.83°, 62.64°, and 74.88°). Using Debye–Scherrer’s formula (Eq. 1), we were able to determine the primary crystallite size of TiO₂.

1 $$D=K\lambda /\beta\;\mathrm{cos}\;\theta$$

where *K* is a constant representing the shape factor (~ 0.9), *λ* is the wavelength of the X-ray source (1.5405 Á), *β* is the full width at half maximum of the diffraction peak, and *θ* is the angular position of the peak. The average crystallite sizes were determined to be around 50 nm for TiO2. Examining the FT-IR spectra (Fig. 1b) of TiO₂ NPs, the anatase phase displays the intense stretching vibrations of Ti–O and Ti–O–Ti bonds, causing the low-frequency bands in the 500–850 cm^−1^ range. The stretching vibration of the surface hydroxyl (− OH) groups on the surface of the TiO₂ nanoparticles can be attributed to a large peak seen between 3500 and 3000 cm⁻^1^.

Analyzing the zeta potential has helped scientists learn about surface charges and determine whether nanoparticles or composites are stable in solutions. Figure 1c and d displays the results showing that particles have a negative charge for TiO₂ (− 26 mV). The stability of particle colloidal dispersions in water was connected to the negative values. There was a considerable degree of dispersion of TiO₂ NPs in the solution, as demonstrated by room temperature zeta potential measurements. The stability of TiO₂ under physiological circumstances was tested using various pH values. More distributed nanoparticles and great stability were observed at a pH of 7.2, according to the data, which corresponds with the physiological conditions.

The spherical morphology of TiO₂ NPs was demonstrated in scanning electron microscopy (SEM), Fig. 1f, taken at various magnifications. This proves that the metal oxide NPs are evenly distributed, with only a minor tendency to clump together. The particle size of TiO₂ NPs was calculated through dynamic light scattering (DLS), where it was documented at around 50 nm.

### Neurobehavioral Tests

#### Open Field Test (OFT)

The association between seven parameters was examined using principal component analysis. Three components were found to have an eigenvalue greater than 1, explaining 81.299% of the total variance. Six items were retained as having a correlation coefficient > 0.6 (Table 2). Component one contains central lines crossed, rearing frequency, and freezing time, whereas lines crossed peripherally, total lines crossed, and total grooming comes separately in two components (2,3). Tables 3 and 4 reveal an increase in the center lines crossed, rearing frequency, and freezing time for the TiO_2_-NP group compared to other groups. On the other hand, ALA restored these measures to control. Further, the total grooming increase in rats treated with TiO_2_-NPs plus ALA related to the TiO_2_-NP and the control groups. Conversely, there was no significant difference between groups for the number of lines crossed peripherally, total lines crossed, and total excretion.

**Table 2:** Loading coefficients > 0.60 for six items of the open field test generated by principal component analysis

Parameters	Component		
	1	2	3
Lines crossed peripherally		0.924	
Lines crossed centrally	0. 870		
Total lines crossed		0.811	
Rearing frequency	0. 835		
Total grooming frequency			0. 827
Freezing time	0.870		
Eigen value	2.671	1.964	1.056
Proportion (%) of *r*^2^	81.299

**Table 3:** Effect of TiO2-NP and ALA administration on adult male rats’ crossed lines and excretion in the open field test

Treatment	Lines crossed centrally (*N*)	Lines crossed peripheral (*N*)	Total lines crossed (*N*)	Total excretion (Freq)
Control	2.00 ± 1.45 ^b^	29.50 ± 2.73^a^	31.50 ± 3.33^a^	3.17 ± 1.92^a^
TiO2-NPs	8.00 ± 1.45^a^	25.17 ± 2.73^a^	33.17 ± 3.33^a^	6.17 ± 1.92^a^
ALA	1.50 ± 1.45^b^	27.17 ± 2.73^a^	28.67 ± 3.33^a^	3.50 ± 1.92^a^
TiO2-NPs and ALA	1.83 ± 1.45^b^	28.33 ± 2.73^a^	30.17 ± 3.33^a^	3.50 ± 1.92^a^
*P*-value	< 0.0001	0.450	0.584	0.387

The data are presented as mean ± standard error. Means bearing different letters (^a–b^) are significantly different in the same column (*P* < 0.05)

**Table 4:** Effect of TiO2-NP and ALA administration on adult male rats’ rearing, grooming and freezing in the open field test

Treatment	Rearing (Freq)	Total grooming (Freq)	Freezing time (s)
Control	14.00 ± 2.01^b^	3.67 ± 3.42^b^	38.17 ± 9.48^b^
TiO2-NPs	22.50 ± 2.01^a^	5.17 ± 3.42^b^	159.33 ± 9.48^a^
ALA	5.83 ± 2.01^c^	8.00 ± 3.42^ab^	41.50 ± 9.48^b^
TiO2-NPs and ALA	15.17 ± 2.01^b^	14.00 ± 3.42^a^	32.67 ± 9.48^b^
*P*-value	< 0.0001	0.032	< 0.0001

The data are presented as mean ± standard error. Means bearing different superscript letters (^a–c^) within the same column are significantly different (*P* < 0.05)

#### Elevated Plus Maze (EPM)

The association between 10 measurements in adult male rats was examined using Principal component analysis. Two components showed an eigenvalue greater than 1, explaining 86.236% of the total variance. One component was removed as having a coefficient < 0.60, and one component with five items was retained, explaining (Table 5) 72.845% of the total variance. Component 1 contains the time elapsed to enter closed arms, the number of open arms entries, the time consumed in open arms, the percentage of time consumed in open arms, and the percentage of open arms entries. Tables 6, 7, and 8 show that the TiO_2_-NP group had the longest time elapsed to enter open arms, while the shortest time elapsed to enter closed arms. Further, it also showed the shortest time expended in open arms, the percentage of time expended in open arms, the number of open arms entries, and the percentage of open arms entries. In contrast, they showed the longest time consumed in closed arms, the percentage of time expended in closed arms, the number of closed arms entries, and the percentage of closed arms entries. Conversely, treatment with ALA restored all measurements close to control values.

**Table 5:** Loading coefficients > 0.60 for five items of elevated plus maze test measurements generated by principal component analysis

Measurements	Component
	1
Time elapsed to enter closed arms	0.855
Open arms entries number	0.741
Spent time in open arms	0.911
Spent time in open arms (%)	0.921
Open arms entries (%)	0.841
Eigen value	7.284
Proportion (%) of *r*^2^	72.845

**Table 6:** Effect of TiO2-NP and ALA administration on adult male rats time elapsed to enter and spent time in open and closed arms of the elevated plus maze test

Treatment	Time elapsed to enter open arms (s)	Time elapsed to enter closed arms (s)	Spent time in open arms (s)	Spent time in closed arms (s)
Control	0.83 ± 1.98^d^	14.00 ± 1.21^a^	115.83 ± 12.46^a^	166.83 ± 13.15^b^
TiO2-NPs	34.33 ± 1.98^a^	1.67 ± 1.21^b^	22.83 ± 12.46^c^	246.67 ± 13.15^a^
ALA	14.00 ± 1.98^c^	12.00 ± 1.21^a^	106.50 ± 12.46^a^	167.50 ± 13.15^b^
TiO2-NPs and ALA	28.83 ± 1.98^b^	13.00 ± 1.21^a^	65.17 ± 12.46^b^	179.17 ± 13.15^b^
*P*-value	< 0.0001	< 0.0001	< 0.0001	< 0.0001

The data are presented as mean ± standard error. Means bearing different superscript letters (^a–d^) are significantly different in the same column (*P* < 0.05)

**Table 7:** Effect of TiO2-NP and ALA administration on adult male rats’ percent of spent time and entries number in open and closed arms of the elevated plus maze test

Treatment	Spent time in open arms (%)	Spent time in closed arms (%)	Open arms entries (%)	Closed arms entries (%)
Control	41.00 ± 4.63^a^	59.00 ± 4.63^c^	55.03 ± 9.98^a^	44.97 ± 9.98^b^
TiO2-NPs	8.60 ± 4.63^c^	91.40 ± 4.63^a^	9.21 ± 9.98^b^	90.79 ± 9.98^a^
ALA	38.84 ± 4.63^a^	61.16 ± 4.63^c^	65.77 ± 9.98^a^	34.23 ± 9.98^b^
TiO2-NPs and ALA	26.65 ± 4.63^b^	73.35 ± 4.63^b^	56.05 ± 9.98^a^	43.95 ± 9.98^b^
*P*-value	< 0.0001	< 0.0001	< 0.0001	< 0.0001

The data are presented as mean ± standard error. Means bearing different superscript letters (^a–c^) are significantly different in the same column (*P* < 0.05)

**Table 8:** Effect of TiO2-NP and ALA administration on adult male rats’ entries number in open and closed arms of the elevated plus maze test

Treatment	Open arms entries (*N*)	Closed arms entries (*N*)
Control	5.00 ± 1.21^a^	3.67 ± 1.24^b^
TiO2-NPs	1.33 ± 1.21^b^	10.50 ± 1.24^a^
ALA	6.33 ± 1.21^a^	3.50 ± 1.24^b^
TiO2-NPs and ALA	4.83 ± 1.21^a^	4.33 ± 1.24^b^
*P*-value	0.004	< 0.0001

The data are presented as mean ± standard error. Means bearing different superscript letters (^a, b^) are significantly different in the same column (*P* < 0.05)

#### Novel Object Recognition Test (NORT)

The principal component analysis determined the association between five measurements during the testing day of the novel object recognition test for adult male rats. Two components had an eigenvalue greater than 1, explaining 83.920% of the total variance. The two components with the four items were retained because they had coefficients > 0.60, as shown in Table 9. Component 1 has three items: investigation number to object C, time of investigation to object C, and discrimination ratio. However, the time of the inquiry to object A comes in component 2.

**Table 9:** Loading coefficients > 0.60 for four items of the novel object recognition test measurements generated by principal component analysis

Measurements	Component	
	1	2
Investigation number to object C	0.774	
Time of investigation to object C	0.697	
Time of investigation to object A		0.666
Discrimination ratio	0.897	
Eigen value	3.054	1.142
Proportion (%) of *r*^2^	83.920	

Table 10 reveals that the investigation number to object C, the time of investigation to object C, investigation number to object A, and discrimination ratio in rats administered TiO_2_-NPs were lower than those of other groups. Furthermore, these were restored to near normal after administration of ALA. On the other hand, the time of investigation for object A was non-significantly different.

**Table 10:** Effect of TiO-2 NPs and ALA administration on adult male rats’ novel object recognition test measurements

Treatment	Investigation to object C (*N*)	Investigation to object A (*N*)	Time of investigation object C (s)	Time of investigation object A (s)	Discrimination ratio
Control	10.17 ± 1.38^a^	1.33 ± 0.97^b^	36.83 ± 4.81^a^	1.83 ± 1.17^a^	0.92 ± 0.09^a^
TiO2-NPs	3.00 ± 1.38^b^	3.67 ± 0.97^a^	15.33 ± 4.81^b^	3.67 ± 1.17^a^	0.65 ± 0.09^b^
ALA	10.83 ± 1.38^a^	2.33 ± 0.97^ab^	36.00 ± 4.81^a^	4.00 ± 1.17^a^	0.80 ± 0.09^ab^
TiO2-NPs and ALA	11.67 ± 1.38^a^	1.00 ± 0.97^b^	34.67 ± 4.81^a^	2.50 ± 1.17^a^	0.84 ± 0.09^a^
*P*-value	< 0.0001	0.051	0.001	0.246	0.037

The data are presented as mean ± standard error. Means bearing different superscript letters (^a, b^) within the same column are significantly different (*P* < 0.05)

#### Morris Water Maze (MWM)

The relationship between the three measurements of the Morris water maze in the spatial learning phase to adult male rats was evaluated using principal component analysis. One component with an eigenvalue greater than 1, explaining 64.79% of the total variance, contains two items with coefficients > 0.60, as shown in Table 11.

**Table 11:** Loading coefficients > 0.60 for two items of the Morris water maze test measurements of the spatial learning section, generated by principal component analysis

Measurements	Component
	1
Trials number reaching the target quadrant	0.734
Spent time in the target quadrant	0.846

Table 12 shows that the elapsed time to escape to the platform was shorter in the ALA group than in other groups. However, the time to reach the target quadrant was longer in TiO2-NP-treated rats than in other groups. On the other hand, the time expended in the target quadrant was longer in ALA-administered rats than in TiO_2_-NP and control groups. Further, the longer time consumed in the target quadrant for TiO_2_-NPs plus ALA than in the TiO_2_-NP group. Moreover, a non-significant variation between treated groups in the number reaching the target quadrant was revealed.

**Table 12:** Effect of TiO2-NP and ALA administration on adult male rats’ Morris water maze test measurements

Treatment	Time elapsed to escape (s)	Time elapsed to reach the target quadrant (s)	Spent time in target quadrant (s)	Trials reaching target quadrant (*N*)
Control	7.875 ± 1.157^a^	4.167 ± 1.217^b^	17.167 ± 1.343^bc^	6.667 ± 0.701^a^
TiO2-NPs	11.667 ± 1.157^a^	8.167 ± 1.217^a^	13.667 ± 1.343^c^	6.167 ± 0.701^a^
ALA	5.717 ± 1.289^b^	1.667 ± 1.217^b^	21.833 ± 1.343^a^	7.500 ± 0.701^a^
TiO2-NPs and ALA	10.158 ± 1.289^a^	2.167 ± 1.217^b^	18.500 ± 1.343^ab^	7.333 ± 0.701^a^
*P*-value	0.006	0.005	0.003	0.523

The data are presented as mean ± standard error. Means bearing different superscript letters (^a–c^) within the same column are significantly different (*P* < 0.05)

### Oxidative Stress Biomarkers

**Fig. 2:**
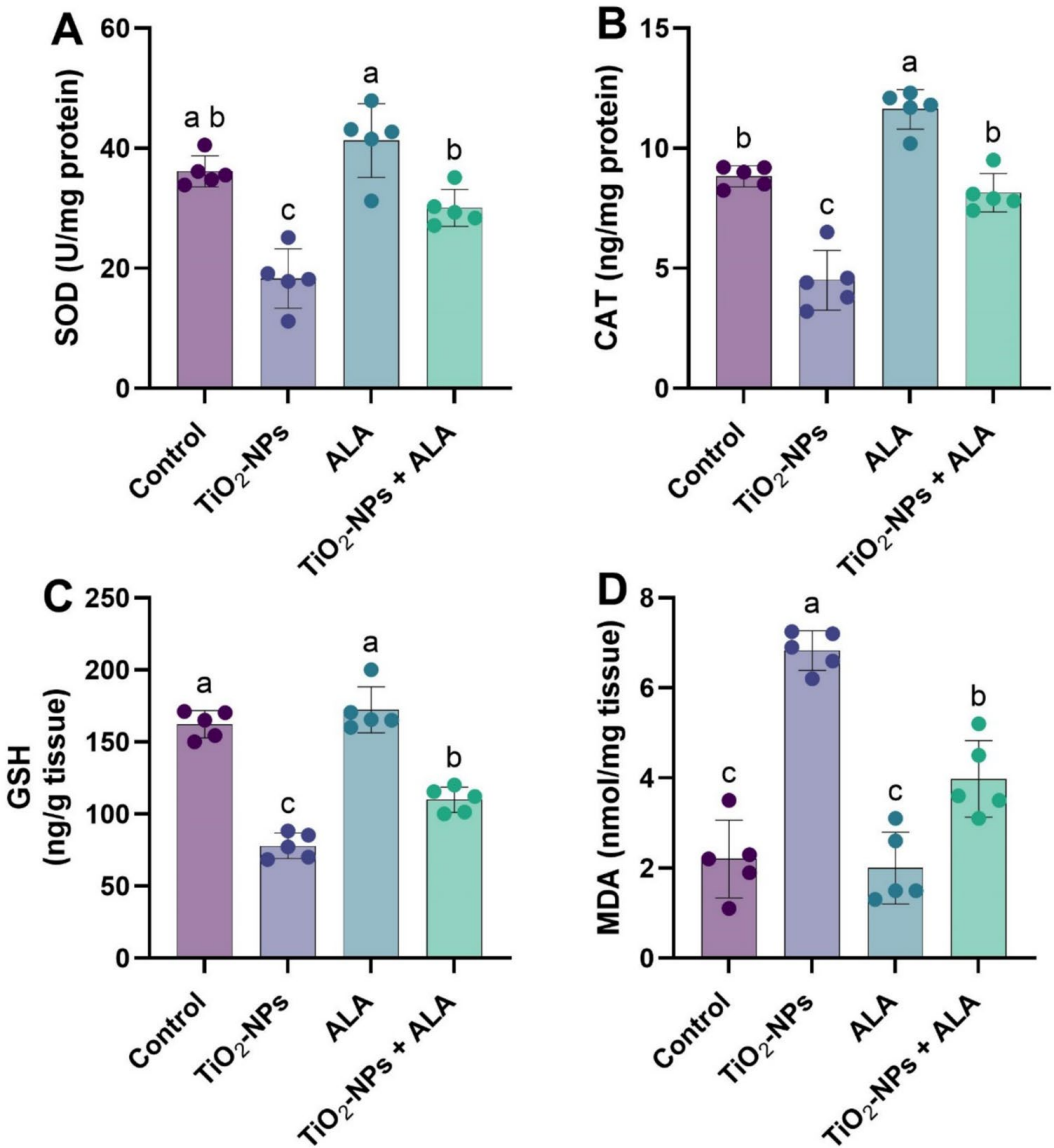
Effect of TiO₂-NPs and ALA on oxidative stress biomarkers in adult male rats. **A** Superoxide dismutase (SOD, U/mg protein); **B** catalase (CAT, ng/mg protein); **C** reduced glutathione (GSH, ng/g tissue); **D** malondialdehyde (MDA, nmol/mg tissue). All values are expressed as mean ± SEM. Different small letters (a–c) indicate statistically significant differences between groups at *p* < 0.05, as determined by one-way ANOVA followed by Duncan’s multiple-range test

The antioxidant status in brain tissue was significantly affected by TiO₂-NP exposure and modulated by ALA co-treatment (Fig. 2A–D). Rats exposed to TiO₂-NPs exhibited a marked decline in SOD, CAT, and GSH levels concerning control (*p* < 0.05). In contrast, MDA level was significantly raised, representing intensified lipid peroxidation. Administration of ALA alone enhanced antioxidant levels significantly compared to the control. Further, co-treatment with ALA and TiO_2_-NPs significantly improved SOD, CAT, and GSH levels and reduced MDA, though not completely to the point, demonstrating partial improvement of oxidative damage.

### Inflammatory and Apoptotic Biomarkers

**Fig. 3:**
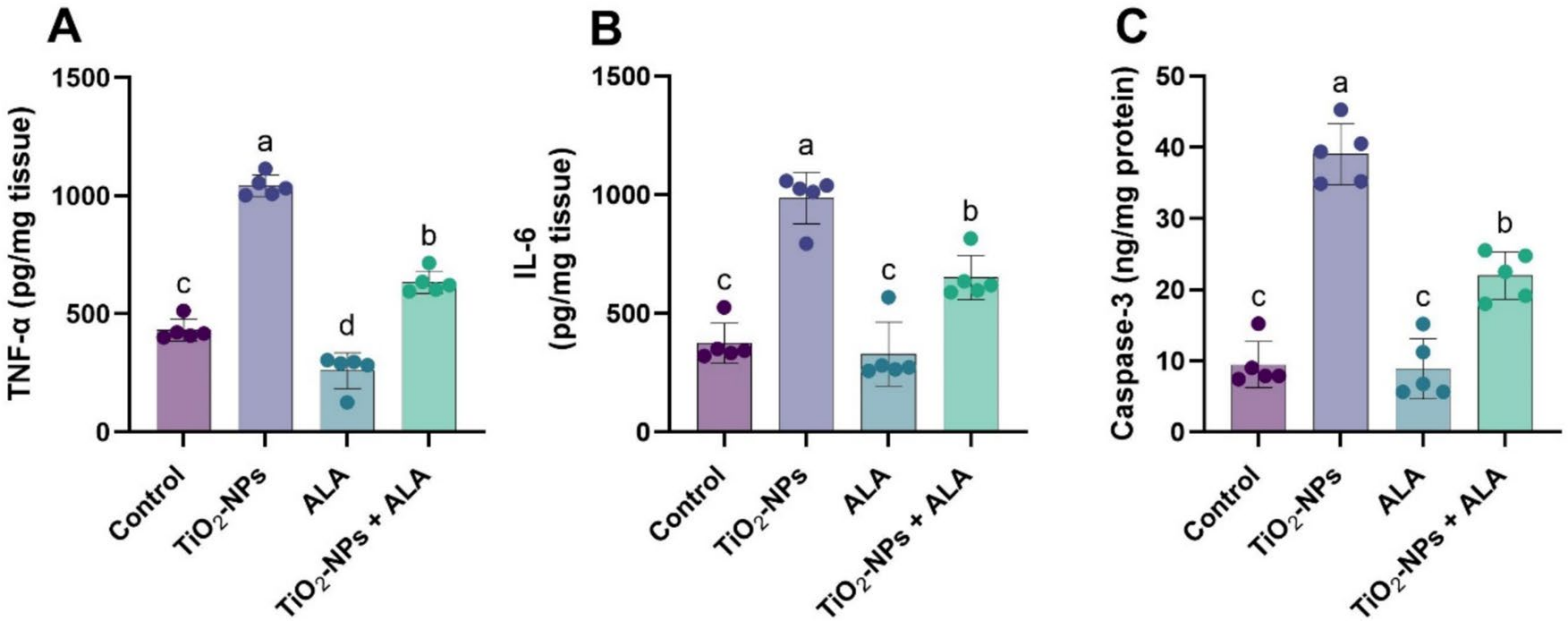
Effect of TiO₂-NPs and ALA on inflammatory and apoptotic markers in adult male rats. **A** Tumor necrosis factor-alpha (TNF-α, pg/mg tissue); **B** interleukin-6 (IL-6, pg/mg tissue); **C** caspase-3 (ng/mg protein). All values are expressed as mean ± SEM. Different small letters (a–d) indicate significant differences between groups at *p* < 0.05, as determined by one-way ANOVA followed by Duncan’s multiple-range test

TiO₂-NPs significantly elevated proinflammatory cytokines TNF-α and IL-6, alongside the apoptotic marker caspase-3, in brain tissue compared to all other groups (Fig. 3A–C). ALA administration alone reduced these inflammatory markers, and co-treatment with ALA in TiO₂-NP-intoxicated rats significantly attenuated the elevated levels. However, these parameters remained higher than in the control and ALA-only groups, suggesting partial ALA mitigation of neuroinflammation and apoptosis.

### Neurotransmitter Levels

**Fig. 4:**
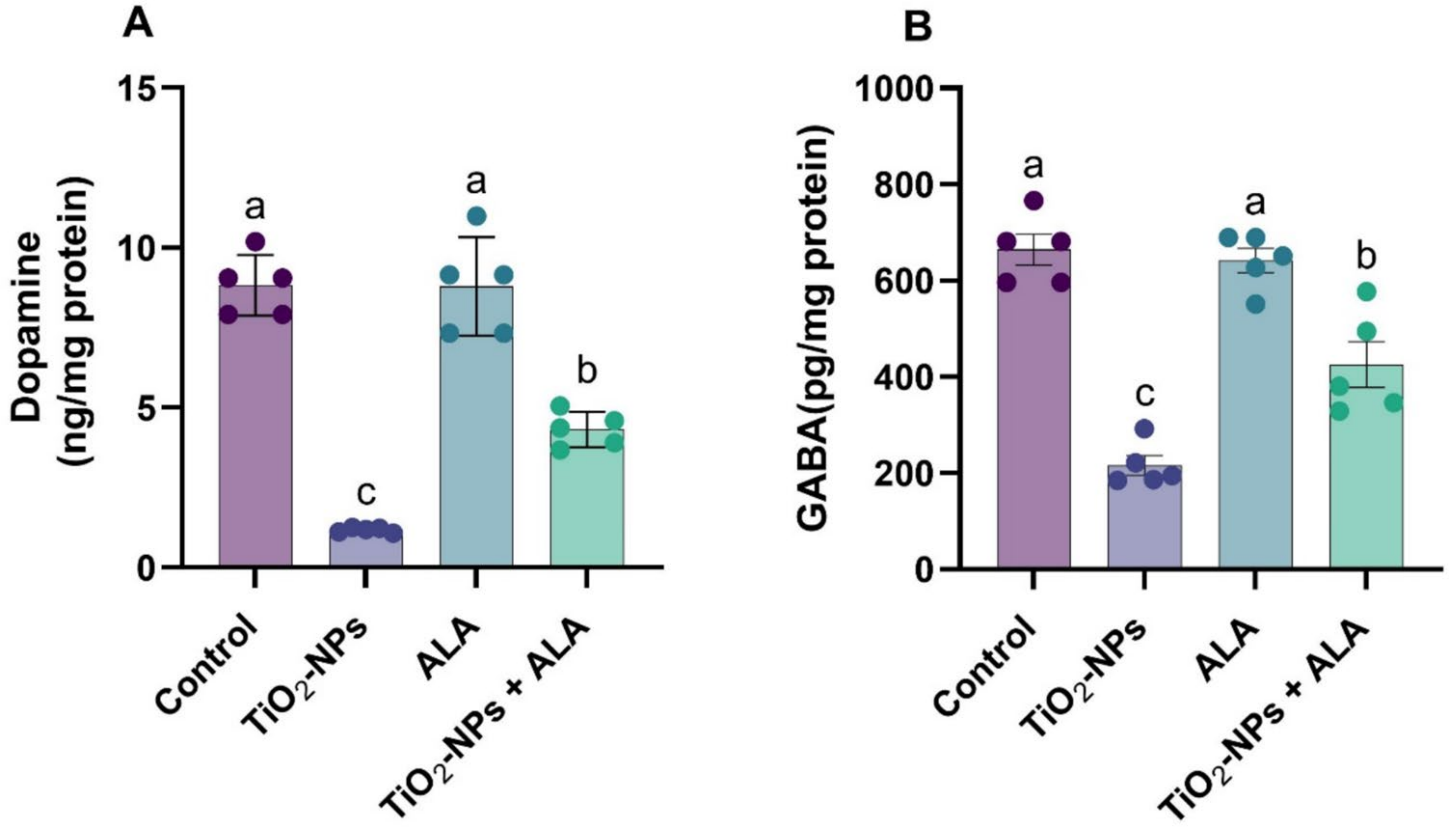
Effect of TiO₂-NPs and ALA on neurotransmitter levels in adult male rats.** A** Dopamine (ng/mg protein); **B** gamma-aminobutyric acid (GABA, pg/mg protein). All values are expressed as mean ± SEM. Different small letters (a–c) indicate statistically significant differences between groups at *p* < 0.05, as determined by one-way ANOVA followed by Duncan’s multiple-range test

As shown in Fig. 4, exposure to TiO₂-NPs led to a substantial decline in dopamine and GABA levels (*p* < 0.05) compared to control, indicative of disrupted neurotransmission. ALA treatment significantly preserved neurotransmitter levels. Co-treatment with TiO₂-NPs and ALA markedly elevated dopamine and GABA associated with TiO₂-NPs alone (*p* < 0.05), although amounts remained below control, showing neurochemical recovery through ALA intervention.

### Gene Expression Analysis

**Fig. 5:**
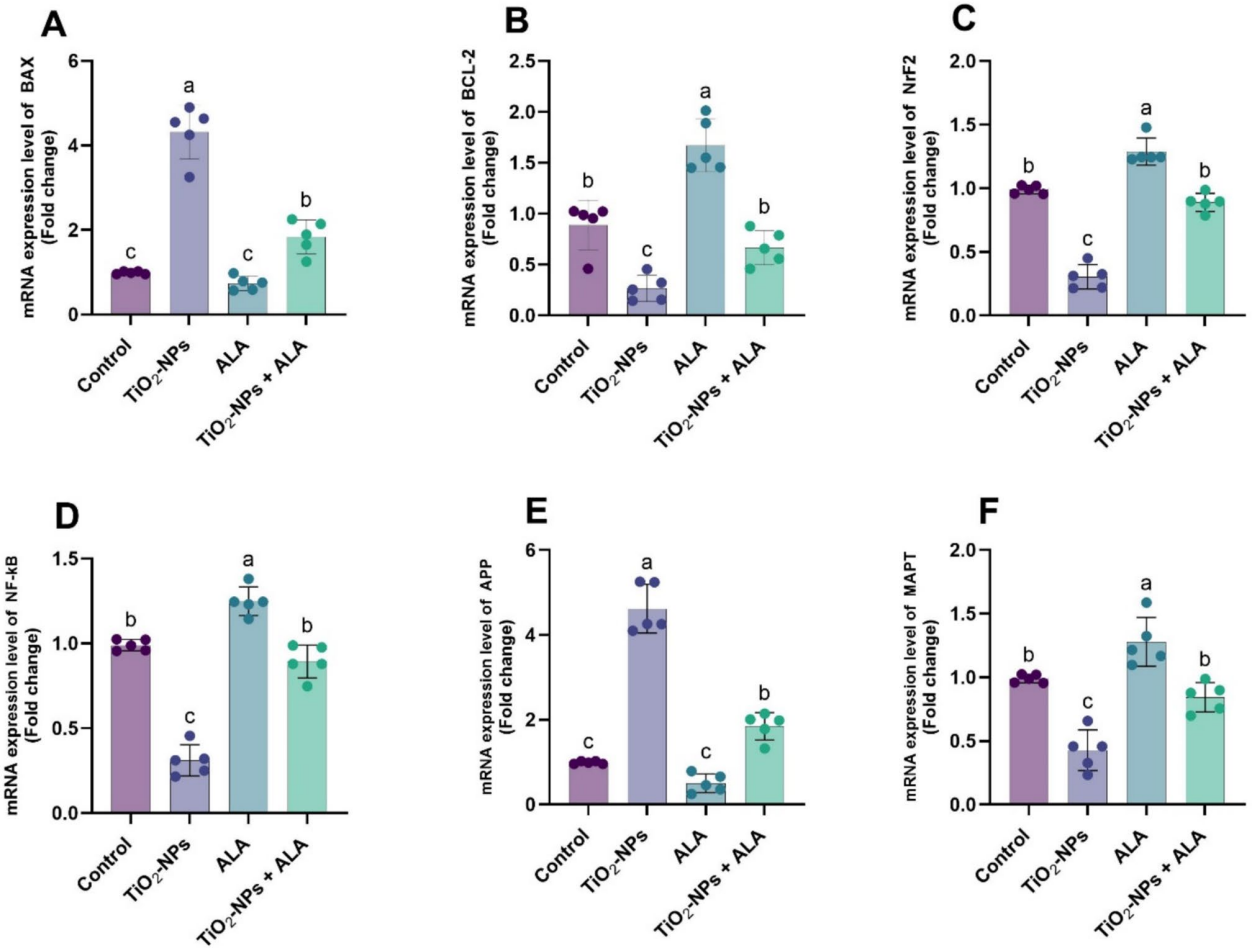
Effect of TiO₂-NPs and ALA on the mRNA expression levels of selected genes in adult male rats. **A** BAX; **B** BCL-2; **C** Nrf2; **D** NF-κB; **E** APP; and **F** MAPT. Gene expression levels are expressed as fold change relative to the control. All values are presented as mean ± SEM. Different small letters (a–c) indicate statistically significant differences between groups at *p* < 0.05, as determined by one-way ANOVA followed by Duncan’s multiple-range test

Gene expression profiles revealed significant modulation by TiO₂-NPs and ALA (Fig. 5A–F). TiO₂-NPs significantly upregulated pro-apoptotic BAX, inflammatory NF-κB, and neurodegenerative markers APP and MAPT. Conversely, anti-apoptotic BCL-2 and antioxidant regulator Nrf2 were downregulated in the TiO₂-NPs group (*p* < 0.05). ALA treatment alone normalized gene expression levels linked to control. In the TiO₂-NPs + ALA group, BAX, NF-κB, APP, and MAPT expression were significantly reduced. In contrast, BCL-2 and Nrf2 expression levels increased in the TiO₂-NP group (*p* < 0.05), indicating that ALA confers molecular neuroprotection by modulating apoptotic, inflammatory, and oxidative stress-related genes.

### Histopathology

**Fig. 6:**
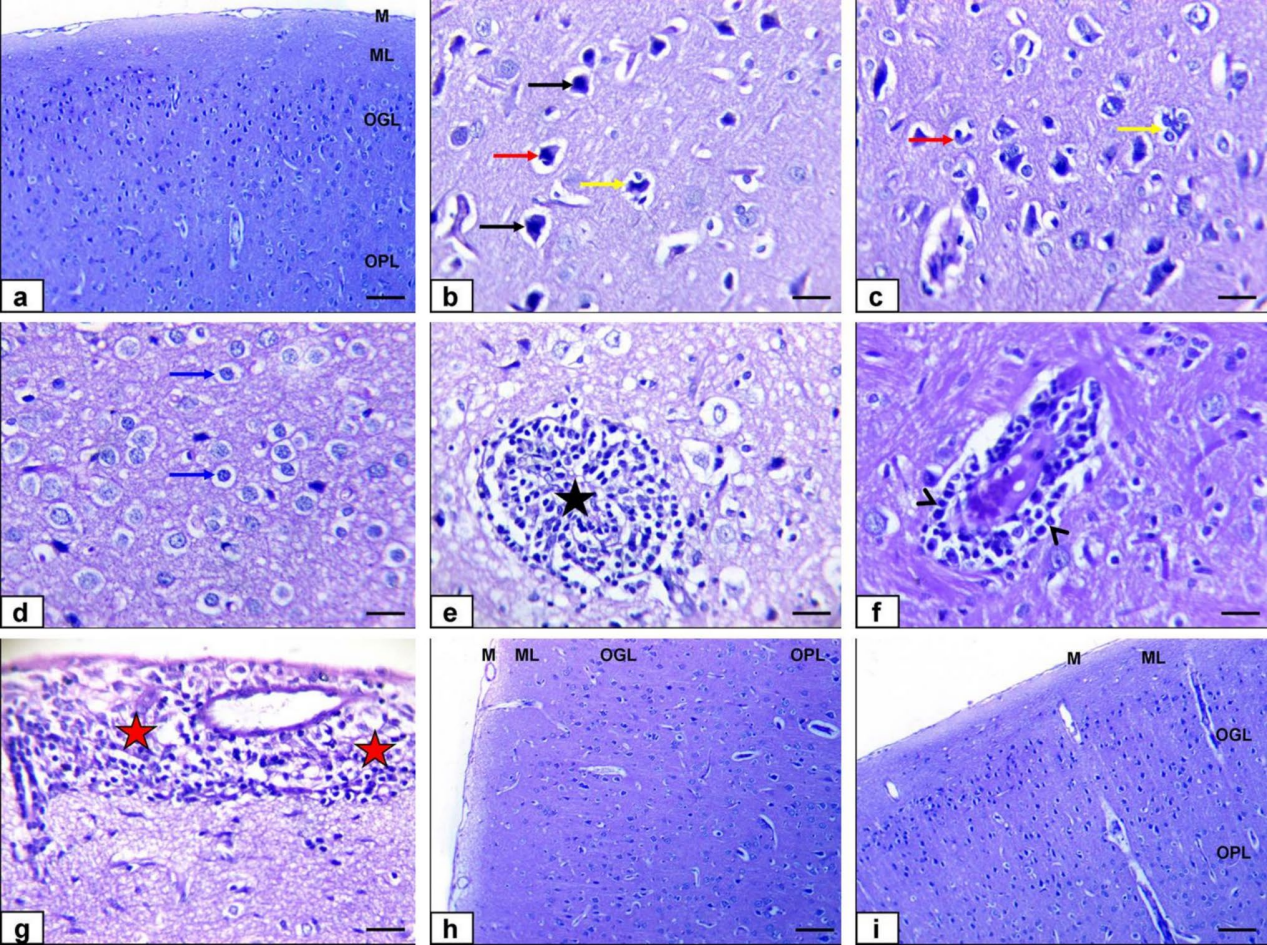
Photomicrographs of the cerebrum of rats stained by H&E **a** Control group showing the normal histological structure of the meninges (M), molecular layer (ML), outer granular layer (OGL), and outer pyramidal layer (OPL) (bar = 100 µm). **b**–**g** TiO2*-*NP-treated group showed that some cerebrum cortex neurons were shrunken. They had hyper-eosinophilic cytoplasm (black arrows) with Satellitosis (yellow arrow) and neuronophagia (red arrow), besides neuronal swelling with neuropil spongiosis (blue arrows), focal gliosis (black star) and perivascular inflammatory cell infiltration in Virchow robin space (black arrowheads) and meninges (red stars) (bar = 50 µm). **h** ALA-treated group showing the normal histological structure of the meninges (M), molecular layer (ML), outer granular layer (OGL), and outer pyramidal layer (OPL) (bar = 100 µm). **i** TiO_2_*-*NPs + ALA-treated group shows the normal histological structure of the meninges and cerebrum cortex layers (bar = 100 µm)

The control and ALA-treated group cerebral cortex presented the normal histological structure of the meninges, molecular layer, outer granular layer, and outer pyramidal layer (Fig. 6a and h). Moreover, the TiO2-NP-treated group exhibited some severely shrunken cerebrum cortex neurons with hyper-eosinophilic cytoplasm with Satellitosis, where the microglial cells encircled a degenerated neuron, then neuronophagia (Fig. 6b and c) besides neuronal swelling with neuropil spongiosis (Fig. 6d) and moderate focal gliosis (Fig. 6). Finally, moderate perivascular inflammatory cell infiltration in Virchow–Robin space and meninges was noticed (Fig. 6f and g). Furthermore, the co-treatment of TiO_2_*-*NPs plus ALA group showed improvement of cerebral histopathological lesions with minimal pyknotic cortical neurons, and almost all histologic sections exhibited normal meninges and cerebrum cortex layer’s histological structure (Fig. 6i).

**Fig. 7:**
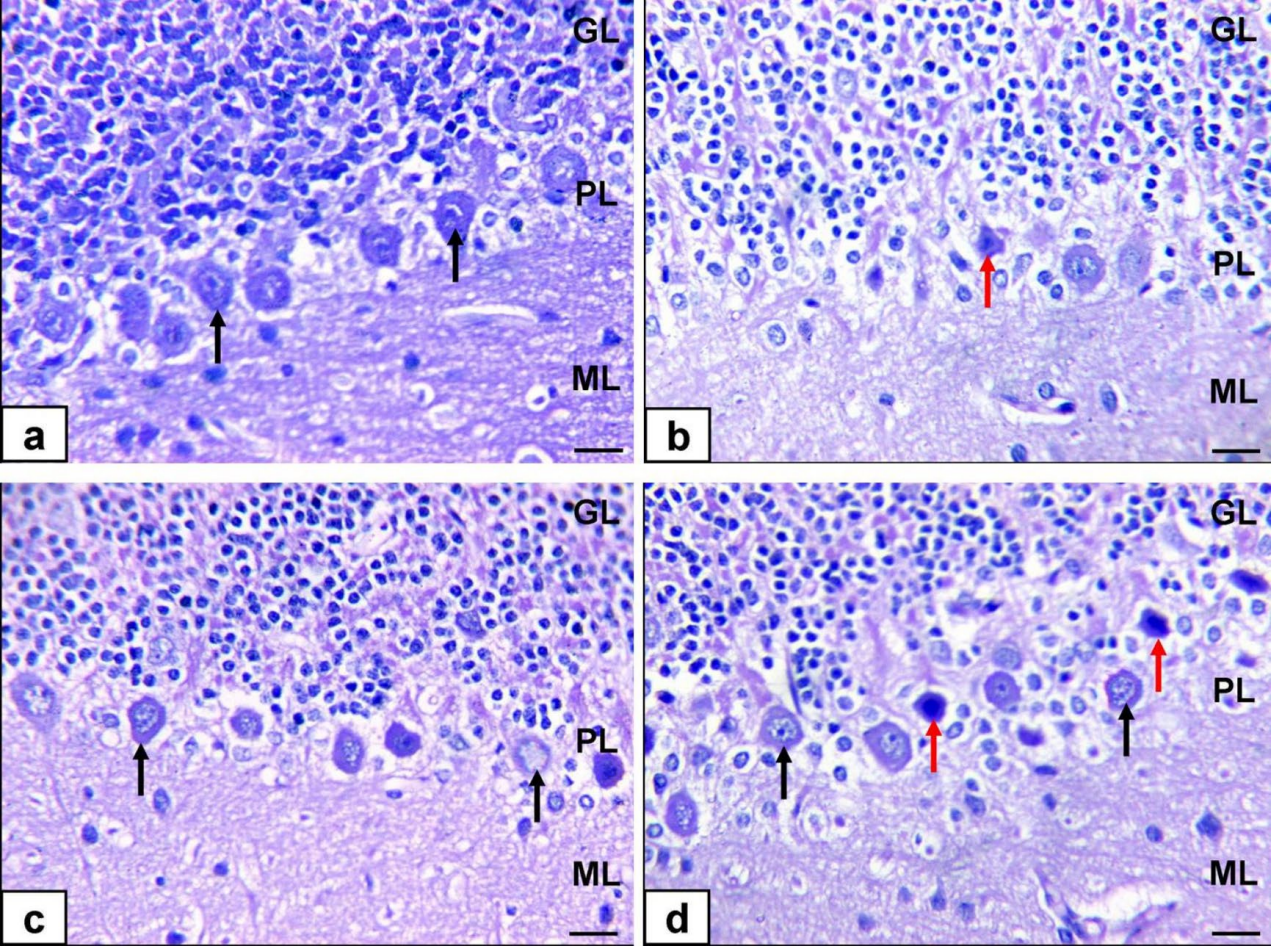
Photomicrographs of the cerebellum of rats stained by H&E. **a** Control group showing the normal histological structure of cerebellar cortex comprised of granular cell layer (GL), the Purkinje cell layer (PL) contain Purkinje cell with their flask-shaped cell bodies, many branching dendrites and a single long axon (black arrows), and molecular layer (ML). **b** TiO_2_*-*NP-treated group showing depletion of the number of granular cell layer beside depletion and pyknotic Purkinje cell (red arrows). (c) ALA-treated group showing the normal histological structure of cerebellar cortex (d) TiO_2_-NPs + ALA-treated group showing a normal histological structure of cerebellar cortex with a mild pyknotic Purkinje cell (red arrows) (bar = 50 µm)

The cerebellar cortex of the control and ALA-treated group displayed normal histological structure of the cerebellar cortex, which comprises the granular cell layer, Purkinje cell layer containing Purkinje cells with their flask-shaped cell bodies, many branching dendrites, and a single long axon, and molecular layer (Fig. 7a and c). Moreover, the TiO_2_*-*NP-treated group exhibited moderate depletion of the number of granular cell layers, besides severe depletion and pyknotic Purkinje cells (Fig. 7b). The TiO_2_*-NP*s plus ALA-treated group showed the normal histological structure of the cerebellar cortex with mild pyknotic Purkinje cells (Fig. 7d; Table 13).

**Table 13:** Incidence and severity of the histopathological lesions of the cerebral and cerebellum cortex in all treated groups

Incidence^1^ and severity^2^ of histopathological lesions																
Groups	Control				TiO2-NPs				ALA				TiO2-NPs plus ALA			
Lesions	−	+	+ +	+ + +	−	+	+ +	+ + +	−	+	+ +	+ + +	−	+	+ +	+ + +
Cerebrum cortex																
Shrunken and had hyper-eosinophilic cytoplasm	5	1	0	0	0	0	2	4	6	0	0	0	4	2	0	0
Satellitosis	6	0	0	0	0	1	2	3	6	0	0	0	5	1	0	0
Neuronophagia	6	0	0	0	0	1	2	3	6	0	0	0	5	1	0	0
Neuropil spongiosis	6	0	0	0	0	1	2	3	6	0	0	0	5	1	0	0
Focal gliosis	6	0	0	0	0	1	3	2	6	0	0	0	5	1	0	0
Perivascular inflammatory cell Infiltration in Virchow–Robin space and meninges	6	0	0	0	0	1	3	2	6	0	0	0	6	0	0	0
Cerebellum cortex																
Depletion of the number of granular cell layer	6	0	0	0	0	2	2	2	6	0	0	0	3	2	1	0
Depletion of the Purkinje cell	6	0	0	0	0	1	2	3	6	0	0	0	3	3	0	0
Pyknotic Purkinje cell	6	0	0	0	0	1	2	3	6	0	0	0	2	3	1	0

^1^Number of rats with lesions per total examined (6 rats per group). ^2^Severity of lesions was graded by estimating the percentage of area affected in the entire section. Lesion scoring: (−) absence of the lesion = 0%; (+) mild = 5–25%; (+ +) moderate = 26–50%; and (+ + +) severe = 50% of the examined tissue sections

## Discussion

Titanium dioxide nanoparticles are extensively utilized in the medical, food, and cosmetic industries due to their exceptional properties, such as whitening and photocatalytic effects. However, previous studies reported that TiO_2_ nanoparticles can cross the blood–brain barrier, accumulate in the brain, and induce neurotoxicity [27]. This raises concerns about exploring protective strategies for mitigating the neurotoxic effects of TiO_2_ nanoparticles. In the present study, we observed the potential ALA neuroprotective effects against neurobehavioral toxicity caused by TiO_2_ nanoparticles in male rats. The principal component indicates a good association between measured parameters in all neurobehavioral tests. Our findings showed that TiO_2_-NPs significantly reduced spatial memory during the Morris water maze as they spent a longer time reaching the target quadrant and a shorter time in it. This was confirmed by histopathological examination. However, the place navigation phase was not significantly affected, which is coherent with the findings of Zhang et al. [28], who stated that a dose of 150 mg/kg bwt TiO_2_-NPs did not result in a significant difference in escape latency in mice; however, our results contradict their non-significant results for time expended in the target quadrant. However, Ze et al. [7] observed that TiO_2_-NPs administered by (2.5, 5, and 10 mg/kg BW) to mice led to a significant increment in escape latency.

On the other hand, in the novel object recognition test, the investigation number and time for object C were lower in rats administered TiO_2_-NPs. Conversely, the TiO_2_-NP group displayed a higher investigation number for object A than the control. This means there was a disturbance in the recognition of the object investigated, which was clarified by the difference in the discrimination ratio. Furthermore, the TiO_2_-NP group performed more crossing to central lines, rearing frequency, and longer freezing time in the open field test. Similarly, the TiO_2_-NP group showed more closed arms entries with longer time expended. However, they had fewer entries and extended time in open arms, with longer elapsed time to enter.

Cui et al. [29] showed that administering TiO_2_-NPs at a dose of 20 mg/kg BW via intraperitoneal injection every 2 days for 30 days in rats led to anxiety-like behaviors. Additionally, Amara et al. [46] presented that rat injected intraperitoneally with TiO_2-_NPs at a dose of (25 mg/kg BW), administered three times over six days, spent less time in the open arms and entered the open arms less frequently during the elevated plus maze test compared to the control group. Furthermore, Younes et al. [30] revealed that administering TiO_2_-NPs intraperitoneally at a dose of 20 mg/kg BW every 2 days for 20 days in rats resulted in fewer entries and a smaller amount of time completed in the open arms of the elevated plus maze, along with an increase in the anxiety index.

Sobhani et al. [31] revealed that administering TiO_2_-NPs orally at doses of 173 and 288 mg/kg BW/day in rats for 7 days decreased locomotor behavior and increased anxiety levels during the open field test.

Our results demonstrated that ALA significantly enhances rats'cognitive abilities and spatial memory. This was indicated by a shorter time elapsed to reach the target quadrant with a longer time expended. These agreed with Khan et al. [32] and Sun et al. [33], who indicated that ALA at 25 and 50 mg/kg BW improved mental ability and memory in mice. Previous studies also revealed that ALA at doses of 50, 70, 100, and 200 mg/kg BW improved cognitive ability, spatial memory, and learning in rats, as demonstrated by an increase in the time consumed in the target quadrant during the Morris water maze [34, 35]. Loss of cognitive ability may arise from neuron damage due to elevated oxidative stress in brain tissue [36]. Furthermore, the ALA restored the number of approaches, time expended to investigate objects, and discrimination ratio in the novel object recognition test to normal in the TiO_2_-NP and ALA groups. Similarly, the central lines crossed, rearing frequency and freezing time in the open field arena, and the number of entries, and time spent in the open and closed arms of the elevated plus maze.

Mahboob et al. [14] reported that co-administering ALA at a dose of 25 mg/kg of body weight per day mixed in the feed along with aluminum chloride in mice for 12 days resulted in an increased discrimination index during the social novelty preference test compared to mice intoxicated with AlCl3 alone. Furthermore, Khan et al. [32] observed that co-treatment of bisphenol A-intoxicated mice with ALA (50 mg/kg BW, i.p.) for 30 consecutive days enhanced short-term recognition memory, as evidenced by the novel object recognition test, when compared to the group exposed only to bisphenol A. Dixit et al. [37] found that administering ALA intraperitoneally at a dose of (70 mg/kg BW) alongside sodium arsenite for 14 consecutive days in rats reduced anxiety in the elevated plus maze compared to rats exposed only to sodium arsenite. Moreover, Wu et al. [38] indicated that administering ALA (0.6 mg/mL) for 8 weeks in drinking water enhanced learning and memory deficits in mice caused by bisphenol A, as demonstrated in the Morris water maze, object placement, and object recognition tests.

ALA’s ability to improve cognitive ability could be attributed to its antioxidative properties, free radical scavenging, lipid peroxidation reduction, and oxidative balance maintenance. Moreover, the improvement in cognition and spatial memory induced by ALA may be due to reduced acetylcholinesterase activity and increased acetylcholine synthesis in the brain, achieved through the activation of choline acetyltransferase [39]. Additionally, ALA’s protective effect could be [40] by inhibiting inflammatory M1 microglia and, consequently, reducing neuroinflammation in the CNS [41]. In the present study, exposure to TiO₂-NPs induced significant oxidative stress, neuroinflammation, neurotransmitter disruption, and gene expression alterations in the brain of male rats. These findings support previous evidence indicating the neurotoxic potential of TiO₂-NPs due to their ability to cross the blood–brain barrier, accumulate in neural tissue, and trigger oxidative and inflammatory responses [8]. Our data showed that TiO₂-NPs significantly decreased the activity of endogenous antioxidants such as SOD, CAT, and GSH while elevating MDA levels, a marker of lipid peroxidation. This is consistent with Cui et al. [29] and Halawa et al. [21], who demonstrated that TiO₂-NPs impair antioxidant defenses, likely due to excessive ROS generation. The co-administration of ALA significantly mitigated these alterations, restoring antioxidant enzyme activities and reducing MDA levels. These effects can be attributed to ALA’s potent-free radical scavenging ability and its role in regenerating other antioxidants, such as glutathione [11]. TiO₂-NPs also significantly elevated the proinflammatory cytokines TNF-α and IL-6 and the apoptotic marker caspase-3. Such elevations indicate the activation of inflammatory cascades and apoptotic pathways, as previously observed by Disdier et al. [42]. These cytokines play a pivotal role in neuroinflammation, which has been linked to neuronal damage and neurodegenerative diseases. Notably, ALA treatment attenuated the expression of these biomarkers, suggesting its anti-inflammatory and anti-apoptotic effects. This aligns with findings from Li et al. [41] and Zaitone et al. [43], who reported that ALA reduces inflammatory signaling by inhibiting M1 microglial activation and decreasing NF-κB-mediated cytokine release.

Furthermore, TiO₂-NPs caused a marked reduction in dopamine and GABA levels, key neurotransmitters responsible for regulating mood, cognition, and motor abilities. The neurotransmitter imbalance observed aligns with previous reports demonstrating that nanoparticle exposure impairs synaptic function and neurotransmission [44]. ALA treatment significantly restored dopamine and GABA levels, which may be due to its modulatory effects on neurotransmitter synthesis and oxidative protection of dopaminergic neurons [32]. Gene expression analysis showed that TiO₂-NP exposure significantly altered the expression of genes involved in apoptosis, oxidative stress, inflammation, and neurodegeneration. Apoptotic imbalance was evident by upregulation of the pro-apoptotic gene BAX and downregulation of the anti-apoptotic gene BCL-2, indicating a shift toward pro-apoptotic signaling [40]. Suppression of Nrf2, a key regulator of antioxidant defense systems, correlated with the observed reductions in SOD and GSH activity, supporting oxidative stress involvement [10]. Increased expression of NF-κB, a master regulator of inflammatory responses, suggests elevated cytokine production and possible glial activation [45]. Additionally, overexpression of APP and MAPT indicates potential Alzheimer-like neurodegeneration, consistent with prior studies reporting β-amyloid deposition and tau pathology following TiO₂-NPs exposure [6, 27, 42].

ALA significantly reversed these gene expression changes, highlighting its neuroprotective potential at the molecular level. Previous studies support ALA’s role in upregulating Nrf2 and BCL-2 while downregulating pro-apoptotic and inflammatory genes [39]. Our findings align with previous research describing the toxicological profile of TiO₂-NPs. Abdou et al. [10] demonstrated that TiO₂-NPs administration in rats significantly increased serum urea, creatinine, and uric acid levels, induced lipid peroxidation, suppressed antioxidant enzymes (SOD, GPx, GST), and elevated inflammatory responses via NF-κB, TNF-α, and HSP-70 upregulation, while inhibiting Nrf2 and HO-1 expression. Similar patterns of oxidative stress and inflammation were observed in our study. The preventive co-administration of ALA in our experiment reduced these deleterious effects. These findings suggest that natural antioxidants, including ALA, can mitigate TiO₂-NP-induced toxicity by enhancing antioxidant defenses and attenuating pro-inflammatory signaling. Our findings suggest that ALA effectively ameliorates TiO₂-NP-induced neurobehavioral toxicity by enhancing antioxidant defenses, suppressing inflammation, preserving neurotransmitter levels, and modulating apoptosis- and inflammation-related gene expression. These results support its potential as a protective agent in preventing nanoparticle-induced neurodegenerative changes.

## Conclusion

In conclusion, this study demonstrates that intraperitoneal administration of titanium dioxide nanoparticles (TiO₂-NPs; 150 mg/kg body weight, on alternate days for 28 days) induced significant neurotoxicity in male rats, characterized by impaired cognitive performance, oxidative stress, neuroinflammation, neurotransmitter imbalance, histopathological alterations in brain tissue, and dysregulation of apoptosis-related and neurodegenerative gene expressions. Co-administration of alpha-lipoic acid (ALA; 50 mg/kg body weight, orally, daily) partially mitigated these toxic effects, improving behavioral performance, antioxidant enzyme activity, neurotransmitter levels, and gene expression profiles, while reducing histopathological damage. These findings underscore the potential of ALA as a neuroprotective agent against nanoparticle-induced neurotoxicity, likely due to its antioxidant, anti-inflammatory, and anti-apoptotic properties. Further studies are warranted to explore its therapeutic potential in preventing nanoparticle-related neurological disorders in humans.

## Data Availability

The data available from the corresponding author on reasonable request.
